# Bacterial Pathogens and Their Antimicrobial Resistance Patterns of Inanimate Surfaces and Equipment in Ethiopia: A Systematic Review and Meta-analysis

**DOI:** 10.1155/2021/5519847

**Published:** 2021-05-13

**Authors:** Teklehaimanot Kiros, Shewaneh Damtie, Tahir Eyayu, Tegenaw Tiruneh, Wasihun Hailemichael, Lemma Workineh

**Affiliations:** Department of Medical Laboratory Sciences, College of Health Sciences and School of Medicine, Debre Tabor University, Debre Tabor, Ethiopia

## Abstract

**Background:**

Hospital-acquired infections have remained a serious cause of mortality, morbidity, and extended hospitalization. Bacterial contamination of inanimate surfaces of the hospital environment and equipment is considered a major contributing factor to the development of several nosocomial infections worldwide. The hospital environment and many devices are an important reservoir of many clinically important bacterial agents including multidrug-resistant pathogens. Therefore, this systematic review and meta-analysis are aimed at investigating bacterial pathogens and their antimicrobial resistance patterns of inanimate surfaces and equipment in Ethiopia.

**Methods:**

An exhaustive literature search was carried out using the major electronic databases including PubMed, Web of Science, MEDLINE, EMBASE, CINAHL, Google Scholar, Cochrane Library, Scopus, and Wiley online library to identify potentially relevant studies without date restriction. Original articles which address the research question were identified, screened, and included using the PRISMA flow diagram. Data extraction was prepared in Microsoft Excel, and data quality was assessed by using 9-point Joanna Briggs Institute critical appraisal tools. Then, data were exported to STATA 16.0 software for analyses of pooled estimation of outcome measures. Estimation of outcome measures at a 95% confidence interval was performed using DerSimonian-Laird's random-effects model. Finally, results were presented via text, figures, and tables.

**Results:**

A total of 18 studies with 3058 bacterial isolates recovered from 3423 swab specimens were included for systematic review and meta-analysis. The pooled prevalence of bacterial contamination of inanimate surfaces and equipment was found 70% (95% CI: 59, 82). Among the Gram-negative bacterial species, the prevalence of ampicillin-resistant *K. pneumoniae* was the highest 80% (95% CI: 78, 92) followed by *Citrobacter* species 78% (95% CI: 57, 83).

**Conclusion:**

This study has shown a high prevalence of bacterial contamination of inanimate surfaces and equipment in Ethiopia.

## 1. Introduction

Hospital-acquired infections (HAIs) are causing the major cause of mortality, morbidity, increased medical costs for treatment, and extended hospitalization. A hospital environment is a major contributing factor to the development of several HAIs worldwide [1]. Contamination of the inert hospital environment, healthcare workers (HCWs), and medical equipment facilitates the rapid spreading of hospital microorganisms from patient to patient, HCW to the patients, and inanimate surfaces to all bodies [2, 3]. Improper equipment sterilization, inadequate decontamination of surfaces, and poor hand hygiene practices of healthcare providers contribute to the cross-transmission of several pathogens including the multidrug-resistant (MDR) strains of bacteria which are responsible for many nosocomial admissions [4, 5].

Bacterial contamination of high-contact communal surfaces (medical charts, bed rails, white coats/scrubs, telephone/cell phones, computer keyboards/mice, and handwashing sink) and medical equipment (blood pressure cuffs, mechanical ventilator, portable radiograph equipment, ultrasound machine, and stethoscopes) is a worrisome healthcare problem to the management and treatments of a critically ill patient [5–7]. Bacterial contamination of inanimate surfaces and equipment is problematic to overcome as it can serve as a reservoir for an unlimited period through a gradual cross-transmission of pathogens and subsequent contact with patients and HCWs at a time of disease management [8]. It can be caused by a range of bacterial (both Gram-positive and Gram-negative isolates) and fungal species [9–12]. A highly virulent pathogen such as *Staphylococcus aureus* (Methicillin-resistant *Staphylococcus aureus* or MRSA), Coagulase-negative *Staphylococci* (CoNS), *Enterococcus* species (vancomycin-resistant *Enterococci*), *Escherichia coli*, *Klebsiella pneumoniae*, *Pseudomonas aeruginosa*, *Clostridium difficile*, and *Acinetobacter baumannii* are capable of harbouring on contaminated inanimate surfaces and medical apparatus [5]. Contributing factors for the transfer of microorganisms from one surface to another may depend on the type of organisms, source of surfaces, surface humidity level, size of inoculum, medical personnel hand hygiene compliance, ward design (overbending), number of colonized patients, and antibiotic stewardship practices [13, 14].

Nosocomial infections can be either emerged from endogenous microflora of the patient during antibiotic therapy or acquired from the exogenous inert environment that plays an important role in the potential reservoir to the microorganism of horizontal infection transmission [5, 15, 16]. In the hospital setting, inanimate surfaces such as mattresses, bed frames, doorknobs, mobile phones, and ophthalmic solutions/eye drops [12] from various wards or units and several medical types of equipment such as stethoscopes, portable radiograph, and ultrasound instruments are the key reservoir for medically important pathogens. Various equipment in the healthcare setting can be utilized in a patient zone for both monitoring and therapeutic purposes. Based on this, it should be decontaminated before and after the patient contact as well as exposure to the environment to mitigate the horizontal transfer of microorganisms from infected patients [14, 17, 18].

The importance of nosocomial infections has grown into the epidemiology and determinants of healthcare-related microbial contamination to implement measures against the rapidly evolving colonization and dissemination of MDR pathogens [3, 14, 19]. This is mainly due to microbial contamination being common among ICU, operating room (OR), adult medical wards, pediatric wards, neonatal intensive care unit, and gynecologic wards [14]. Lack of regular cleaning and disinfection practices is believed as the main factor for the spread of HAIs. Besides monitoring the microbial quality of medical equipment, the regulation of indoor air bacterial load of the healthcare rooms has a significant role in the health of occupants [20–22]. This is because the bacterial pathogens can survive and remain viable on inert surfaces and/or equipment due to their ability to form biofilms which allow withstanding against jeopardy environmental conditions around their niche. Otherwise, factors such as surface porosity and humidity shall exist. Besides, it enhances the pathogens to adapt to environmental stress and selected pressures at its vicinity [2, 3, 23].

Decontamination with the use of physical or chemical means to remove and inactivate the contaminant pathogens from the surfaces of the hospital environment in order to provide a safe environment for handling of the patient is essential [12, 24, 25]. Not only decontamination processes but also disinfection, cleaning, and sterilization are very important steps to be done for a reusable item safe for further medical use. Failure to properly adhere to these techniques toward the inanimate surfaces and equipment not only is a risk linked with a break of host barriers but also is a risk for person-to-person transmissions and transmission of environmental pathogens [26, 27].

In general, understanding the epidemiology and determinants of microbial contamination at a country level is fundamental to reinforce effective decontamination, disinfection, cleaning, and sterilization methods to mitigate microbial dissemination and systematic surveillance of MDR pathogens in Ethiopia. Although there are some individual studies concerning bacterial contamination of inanimate surfaces and equipment in Ethiopia, a systematic review and meta-analysis study regarding bacterial pathogens and their antimicrobial resistance patterns of inanimate surfaces and equipment in Ethiopia is unavailable as far as our knowledge goes. Therefore, this study is aimed at performing a systematic review and meta-analysis to estimate the overall prevalence of bacterial contamination of inanimate surfaces and equipment in Ethiopia.

## 2. Methods

### 2.1. Study Design, Setting, and Period

This systematic review and meta-analysis study was conducted in Ethiopia. It is the second-most populous country next to Nigeria in the Africa continent with a current total population greater than 115 million (https://worldpopulationreview.com/countries/ethiopia-population).

Any laboratory-based studies that address the outcome of interest in light of the concept of bacterial contamination of inanimate surfaces and equipment conducted using the standard microbiological protocols from the Ethiopian population were systematically studied. Laboratory-based studies conducted in different public and private health intuitions (hospitals, health centres), as well as wards or units including medical wards (MW), pediatric wards (PW), orthopaedic wards (OPW), surgical wards (SW), gynecologic wards (GW), emergency wards (EW), intensive care unit (ICU), and neonatal intensive care unit (NICU), were studied. Consequently, a systematic review and meta-analysis study was conducted to sum up the pooled prevalence of bacterial contaminants isolated from different contaminant reservoirs and their drug resistance patterns reported from the various regions of Ethiopia. Any relevant studies addressing the research objective were considered for screening regardless of the study period provided that any updates till the date of the manuscript submission for publication were considered.

### 2.2. Literature Search Strategy

An exhaustive literature search strategy toward studies reporting the prevalence of bacterial contamination of inanimate surfaces and equipment was conducted for grey and peer review literature with no date restrictions. Electronic database search engines such as MEDLINE, PubMed, Cochrane Library, Scopus, Google Scholar, EMBASE, CINAHL, Wiley online library, Index Medicus, Africa Journals Online, Clarivate, medRxiv, bioRxiv, and Web of Science were exhaustively searched to identify potentially published relevant studies. Besides, expert consultation, reference tracing of potential full-text articles, preprints, and conference proceedings were carefully assessed to complete the search strategy. Additional data was sought even from the authors to complete the information through email contact, especially for inaccessible/full of charge original research articles. Further, regular alerts were established to few selected databases like PubMed and Google Scholar to update the search strategy before the publication of the article. Moreover, Google and other internet search engines were used to search for additional web-based or electronic materials. Hence, the searches were rerun just before the final data analyses.

Keywords and controlled vocabularies are used for the search; the relevant materials used for the review were selected by the authors. As a result, keywords were developed following the medical subject heading (MeSH) search strategy. The Boolean operators (AND, OR, and NOT) and wild cards (“∗”) were customized by the authors based on the research questions of the outcome measures. Accordingly, filters like language, year, subject, and article type as well as helpful search tags were used. The literature search strategy was based on the following keywords and phrases: “microbial contamination”, “bacterial contamination”, bacterial contamination of inanimate surfaces OR “bacterial contamination of equipment”, “microbial contamination of inert hospital environment” OR “prevalence of bacterial contamination of equipment, inanimate surfaces” AND “Ethiopia”, “indoor air bacterial load determination”, “ward bacterial contamination”, OR “equipment contamination” AND “Ethiopia”. Besides, searching using specific bacterial species like “*Staphylococcus aureus*” OR “*E. coli*” OR “*Acinetobacter baumannii*” AND “ICU ward contamination” AND “Ethiopia” was made.

### 2.3. Operational Definition

#### 2.3.1. Medical Equipment

Medical equipment is any device including a sphygmomanometer, stethoscope, and thermometer used for the diagnosis and therapeutic purposes for hospitalized patients in pediatrics, ICU, neonatal intensive care unit (NICU), and surgical, medical, gynecology, and orthopaedic wards/units [5, 14]. Nonmedical devices that harbour microbes also include computers and HCW's cell phones as well as other equipment found in the hospital environment that has contact with HCW.

#### 2.3.2. Inanimate Surfaces

These are a surface of the inert hospital environment and the surface of the material used during patient treatment and management such as bedside tables, mattress, computers, computer standing tables, ophthalmic solutions or multidose eye drops, white coats/scrubs, telephone/cell phones, and handwashing sink [5, 12, 28].

#### 2.3.3. Indoor Air

This is the air inside the rooms, wards, and units during laboratory investigation [3].

#### 2.3.4. Settle Plate or Passive Air Sampling

Petri dishes containing blood agar plates are left open to air for a given period; then, microbes carried by inert particles fall onto the surface of the nutrient with an average deposition rate of 0.46 cm/s being reported [17, 29].

### 2.4. Inclusion and Exclusion Criteria

Before identifying appropriately published relevant full-text articles either in local or international journals, a selection criteria checklist for study eligibility was developed by the authors.

#### 2.4.1. Inclusion Criteria

All studies which met the following criteria were included in the review process: [1] studies that reported the prevalence of bacterial contamination from inanimate surfaces and/or equipment, [2] studies published in English language but conducted only in Ethiopia at any date, [3] studies conducted using the standard bacteriological techniques (i.e., using swab method or settle plate sampling method following a 1/1/1 schedule. That means sterile Petri dishes containing 5% sheep's blood agar were left on the air for 1 hour and 1 meter above the floor as well as 1 meter away from the wall [17, 29].), [4] studies accurately reporting the swab culture growth rate for bacterial isolates and their drug susceptibility/resistance tested against selected commercially available drugs used for the treatment of HAI based on the clinical laboratory standard institute (CLSI) document [30] [5], all relevant free-of-charge full-text original research articles, and [6] studies reporting the prevalence from nonmedical equipment like the mobile phone of HCWs, Ethiopian currency notes or coins, computers, and bus surfaces. In addition, any online freely available (preprint and not peer-reviewed) materials like dissertations (MSc, PhD) were also included. At the same time, records retrieved from instructional repository digital libraries (private and public institutions) were included.

#### 2.4.2. Exclusion Criteria

The study was excluded for the following reasons: [1] inaccessible or irretrievable full-text articles after requesting from the corresponding authors via email or research gate account; [2] review, commentaries, letters to the editor, conference proceeding, and abstracts; [3] studies that report bacterial contamination from environmental entities (soil, lake, river, and hospital effluent water); [4] reports from food items (dairy products, meat, coffee, fruits, vegetables, and cereals); [5] studies done on microbiological assessment of safety, quality of drinking water, and nonalcoholic beverages; [6] studies on animal microbial colonization; [7] studies having mixed sample sources and results (swab, water, and food); and [8] microbial contamination of the inert environment and/or devices due to microbial toxins such as aflatoxins and mycotoxins. Besides, studies were excluded if there is incomplete information to address the primary goal of the research question. For example, studies with insufficient/vague outcome measures after review by peer reviewers independently were discarded. Not surprisingly, fungal contamination of inanimate surfaces and equipment was automatically excluded from the entire review process.

### 2.5. Data Screening, Extraction, and Management

To enhance screening, online records from various databases and directories were exported appropriately to ENDNOTE reference software version 8.2 (Thomson Reuters, Stamford, CT, USA). Then, the records were merged into one folder to identify and remove duplicate articles with the help of ENDNOTE or manual tracing as there are several possibilities of citation styles per article. Thereafter, a couple of authors Teklehaimanot Kiros (TK) and Tegenaw Tiruneh (TT) independently screened the title and abstracts of each article based on the predefined eligibility criteria as mentioned above (inclusion/exclusion criteria). Records that passed the screening phase were further subjected to eligibility assessment of full-text articles according to the critical appraisal checklist for systematic reviews and research syntheses [31]. For this, three authors, TK, Tahir Eyayu (TE), and Shewaneh Damtie (SD) independently collected full-text articles and evaluated their eligibility for meta-analysis. In case of discrepancy among authors, Wasihun Hailemichael (WH) and Lemma Workineh (LW) were assigned to facilitate rechecking the review process (primarily for accuracy and consistency) until mutual or anonymous consensus was reached to any arisen disagreement between or among the authors. The data extraction was performed by peer reviewers (TK with TE and TT with SD) who independently extracted all relevant articles using a standardized and pretested format prepared in Microsoft Excel. The authors designed a data extraction form adopted from Cochrane collaboration and Preferred Reporting Items for Systematic Reviews and Meta-analyses (PRISMA, 2009 Checklists) as shown (Supplementary file 1: Table S1: PRISMA checklists). Lastly, a checklist is customized into our study protocol to address all research questions and outcome measures. The data extraction format included principally study ID, first author and reference, study design, study setting, publication year, study site in the country, sample size, sampling technique, specimen collection method along with a source of contamination (inanimate surfaces and equipment), the prevalence of outcome of interest, types of bacterial isolates, and antimicrobial resistance patterns. In cases of insufficient/incomplete data, the authors independently reviewed the full text of the article for further information and clarification. Any inconsistencies were resolved through discussion until a consensus is reached among the authors assigned for data retrieval. Thereafter, extracted data from each article were summarized into Microsoft Excel and spreadsheet. The list of references and laboratory data for each study were carefully cross-checked to ensure no redundancies coexisted. Duplicate studies were excluded; otherwise, they provide additional outcome measurements based on the review objective. Finally, the study selection process was presented using the PRISMA flow diagram for all studies reviewed, screened, and included in the quantitative synthesis or meta-analysis as described previously [32]. Finally, a total of 18 eligible original articles were included in this meta-analysis.

### 2.6. Outcome Measurements

This systematic review and meta-analysis study from Ethiopia has three major outcomes. The first outcome of interest was to determine the overall pooled prevalence of bacterial isolates (culture positive) recovered from inanimate surfaces and devices summarized from the different regions in Ethiopia. The second outcome was to identify and estimate the pooled proportion of the etiologic agents causing inanimate surface and equipment contamination. The third outcome measure of the study was to summarize the drug resistance patterns of the pathogens recovered from the various sources of contaminants across the regions of Ethiopia.

### 2.7. Quality Assessment

Critical appraisal of the studies was made by assigned reviewers to ensure the accuracy and consistency of data. The quality of studies was assessed using standard critical appraisal tools prepared by the Joanna Briggs Institute (JBI), at the University of Adelaide, Australia [33]. The main objective of the appraisal was to carefully assess the methodological quality of studies, the possibility of bias in its design, and the extent to which the statistical analysis and data synthesis are addressed. The JBI appraisal checklist for prevalence studies has nine important questions. The questions (Q1-Q9) primarily focus on the appropriateness of the sampling frame to address the target population, appropriateness of sampling techniques, adequacy of the sample size, the details of study subjects and setting, the depth of statistical analysis, the presence of valid methods to identify the condition, the extent it is measured as per the standard, the appropriateness of the methods, and adequacy of the response management. The critical appraisal was also conducted to evaluate the internal (systematic error) and external validity of studies thereby reducing the risk of biases among individual studies. In all cases, scores of the two authors (TK and TT) in consultation with a third author (TE) in case of discrepancy (between the two authors' appraisal result) were taken for a final decision. Total scores ranged between 0 and 9. Finally, studies with the number of positive responses (yes) greater than half of the number of checklists (i.e., a score of five and above) were included in the systematic review and meta-analysis.

### 2.8. Assessment of Publication Bias

Publication bias assessment was conducted using a funnel plot. For this study, the presence of publication bias was examined using a funnel plot and Egger's test. Upon the visual inspection of the funnel plot, the asymmetrical distribution of studies on the funnel plot might suggest the presence of publication bias due to the small study effect [34, 35].

### 2.9. Data Synthesis, Analysis, and Reporting

The extracted data were imported from Microsoft Excel to STATA software for the pooled estimation of outcome measures. Data manipulation and statistical analyses were performed using STATA software version 16 (College Station, Texas, USA) [36]. DerSimonian-Laird's random-effects model was applied to estimate the overall pooled prevalence of bacterial contamination at a 95% confidence level. The model is recommended to adjust for variability in the presence of heterogeneity among studies [37]. Sensitivity analysis and subgroup analyses were also conducted to minimize the degree of heterogeneity across studies. Meanwhile, heterogeneity was checked using *I*^2^ test statistics. *I*^2^ test statistics is the preferable and more reliable test to measure the variability across the studies. It ranges between 0 and 100%. *I*^2^ ≤ 25% suggested more homogeneity, 25% < *I*^2^ ≤ 75% suggested moderate heterogeneity, and *I*^2^ > 75% suggested high heterogeneity [38]. The subgroup analysis was carried out based on the study region and methods of sample collection. This reduces the random inconsistency between the point estimates of the primary study. Finally, all statistical tests with *p* values less than 0.05 and corresponding 95% CI were considered statistically significant. The results of the findings were presented by texts, summary tables, and figures (forest plots).

## 3. Results

### 3.1. Literature Search

**Figure 1:**
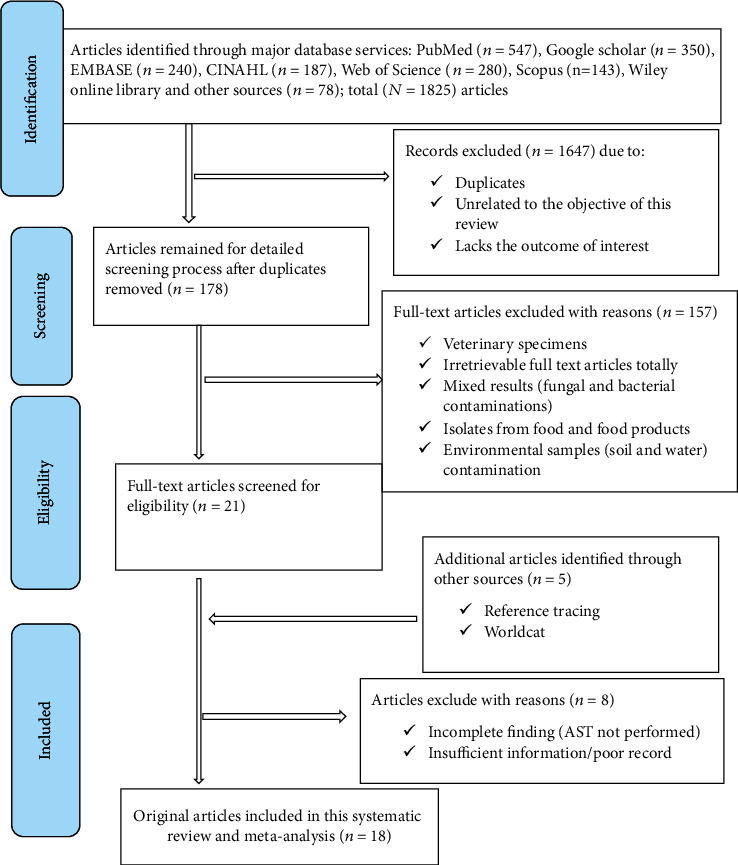
PRISMA flow diagram for searched, screened, and included studies.

A comprehensive literature search was made in major electronic database engines including Google Scholar, PubMed, MEDLINE, Web of Science, EMBASE, and CINAHL and yielded a total of 1825 publications. Among the total, 1647 records were discarded due to duplications assessed by ENDNOTE and/or manual tracing, unrelation to the objective of the review question, and being simply a qualitative study. The remaining articles that were subjected to a detailed screening process (*n* = 178) were further thoroughly assessed to sufficiently meet the primary outcome measures satisfactorily and unambiguously. Among these, *n* = 26 were screened for the eligibility of the full-text articles. Based on the predetermined inclusion and exclusion criteria, the removal of articles with reasons such as reports from veterinary specimens, food, and food products and environmental entities (soil and water), as well as incomplete results concerning the research objective, was made (Figure 1). In this regard, 8 studies were removed due to failure to meet the inclusion criteria for our study. Consequently, only a total of 18 original full-text articles addressing the primary outcome measures sufficiently and unambiguously were included in this systematic review and meta-analysis study.

### 3.2. Quality Assessment

In this meta-analysis, quality assessment for all included laboratory-based cross-sectional studies was conducted based on the JBI critical appraisal checklist. The checklist has nine fundamental questions (Q1-Q9) with total scores ranging from zero to nine. Studies with average quality scores ranging between five and nine were included in the systematic review and meta-analysis. As a result, 18 studies that have fulfilled the inclusion criteria and rigorous appraisal based on the JBI tools were included for systematic review and meta-analysis (Table 1).

### 3.3. Characteristics of Included Studies

The year of publication of the included studies was ranged from 2013 [24] to 2020 [19]. Four out of the total articles were published in the year 2019 [4, 28, 40, 45]. Among the total of 18 included studies, three were from Oromia [3, 24, 42], Amhara [4, 29, 39], Tigray [28, 45, 46], Sidama [41, 48, 50], and southern Ethiopia [43, 44, 47] regions. Unfortunately, only two studies [19, 49] were included from Addis Ababa (central Ethiopia). All included studies were done by using the laboratory-based cross-sectional study design. Similarly, 6/18 (33%) were conducted using a simple random sampling technique. Likewise, both settle plate (air sampling) methods and swab methods (surface swabbing) were used for specimen collection for bacterial contamination of inanimate surfaces and equipment. However, only two studies [29, 46] were conducted using both techniques. In this meta-analysis study, a total of 3423 samples were used and 3058 isolates were detected as summarized in Table 2.

A total of 3058 bacterial isolates were recovered from 3423 swab specimens with 2273 culture-positive growths. In the meantime, the number of isolates, swab samples, and positive bacterial culture ranges from 66 to 344 [39, 45], 78 to 422 [3, 4], and 41 to 243 [4, 41], respectively. Regarding the bacterial profile of inanimate surfaces and equipment contamination, *S. aureus*, CoNS, *E. coli*, *P. aeruginosa*, *K. pneumoniae*, and *Citrobacter* spp. were the most common clinically relevant pathogens. The number of clinically relevant Gram-positive bacterial species (*S. aureus* and CoNS) and Gram-negative bacterial species (*E. coli*, *P. aeruginosa*, *K. pneumoniae*, and *Citrobacter* spp.) that were recovered from swab samples of different contaminant reservoirs (wards/units, HCW fomites, and air) is summarized in Table 3.

### 3.4. Study Outcome Measures

#### 3.4.1. Primary Outcome Measures: Prevalence of Bacterial Contamination

**Figure 2:**
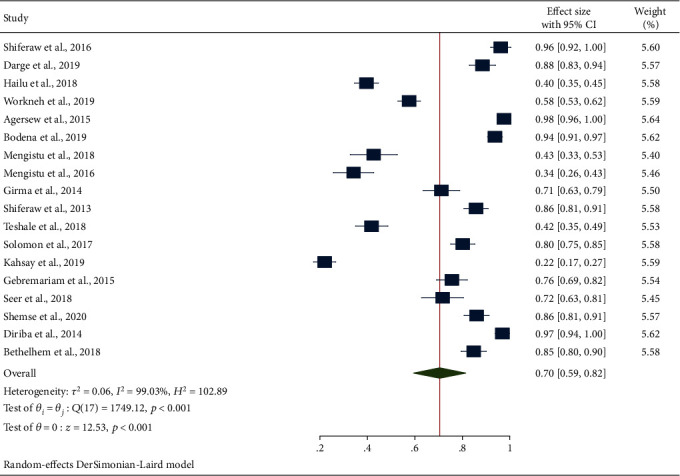
Forest plot depicting the overall prevalence of bacterial culture positivity obtained from swabs of inanimate surfaces and equipment contamination in Ethiopia, 2013-2020.

In this study, a total of 3058 isolates with 2273 positive cultures were found from 3423 swab specimens. The pooled prevalence of bacterial contamination of inanimate surfaces and equipment was found to be 70% (95% CI: 59, 82) as shown in Figure 2.

#### 3.4.2. Subgroup Analysis

**Figure 3:**
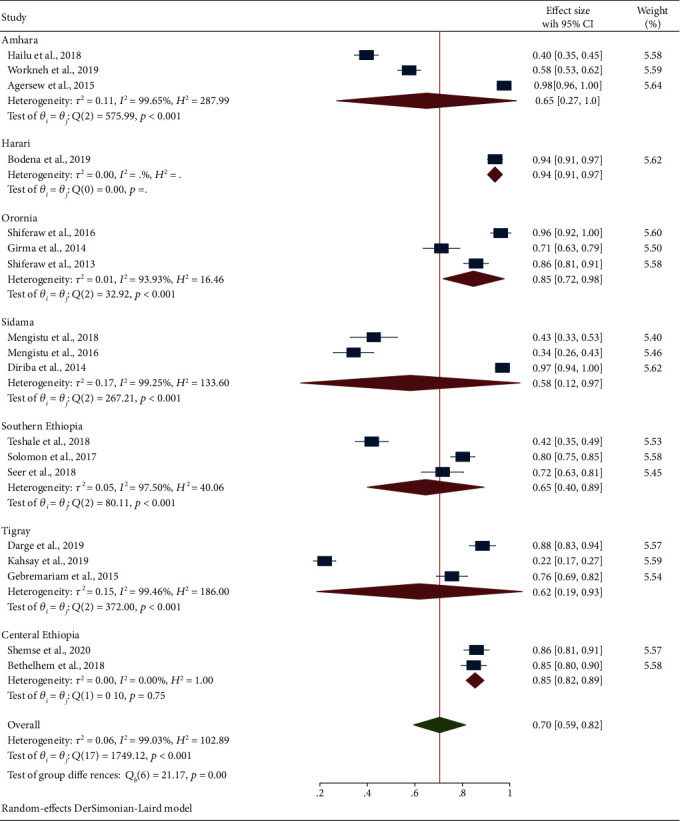
Forest plot depicting the subgroup analysis based on study regions for bacterial culture positivity obtained from swabs of inanimate surfaces and equipment contamination in Ethiopia, 2013-2020.

**Figure 4:**
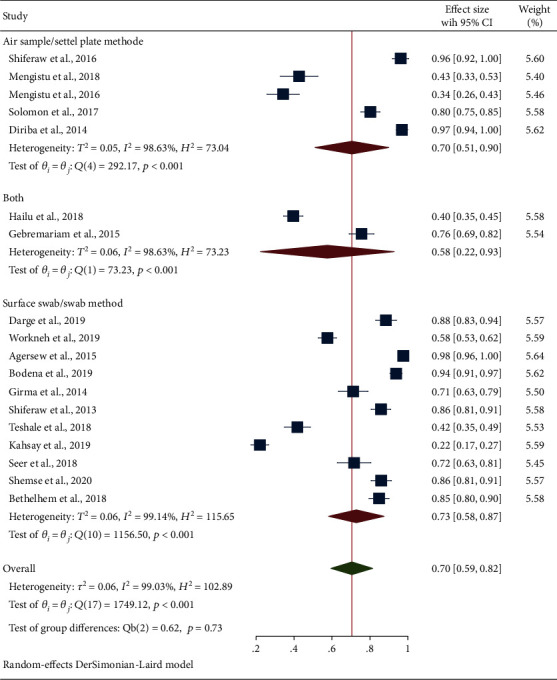
Forest plot depicting the subgroup analysis-based specimen collection methods for the swabs of inanimate surfaces and equipment contamination in Ethiopia (2013-2020).

Subgroup analysis based on the study region has shown that the Oromia (85%, 95% CI: 72, 98) and central Ethiopia (85%, 95% CI: 82, 89) regions ranked the first followed by the Amhara region (65%, 95% CI: 27, 100) and Tigray region (62%, 95% CI: 19, 93). Also, the overall prevalence of culture positivity obtained from bacterial contamination of inanimate surfaces and equipment and their corresponding subgroup analysis based on study regions were depicted using the forest plot as shown in Figure 3. Furthermore, subgroup analysis based on the specimen collection methods was conducted. Based on the methods, the pooled estimate of the surface swab/swab method was slightly higher (73%) than the settle plate method (70%) as depicted in Figure 4.

#### 3.4.3. Secondary Outcome Measures: Estimation of Bacterial Pathogens

**Figure 5:**
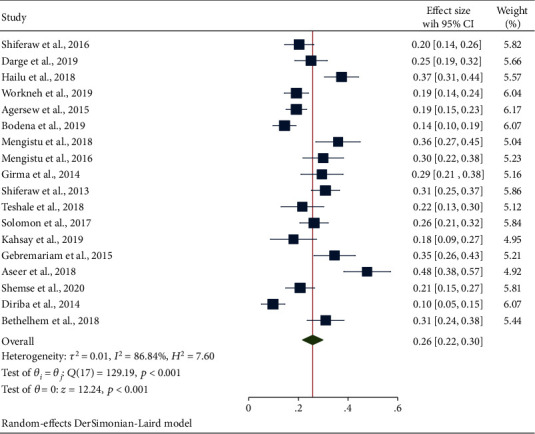
The forest plot showing the prevalence of S. aureus from swabs of inanimate surfaces and equipment in Ethiopia.

**Figure 6:**
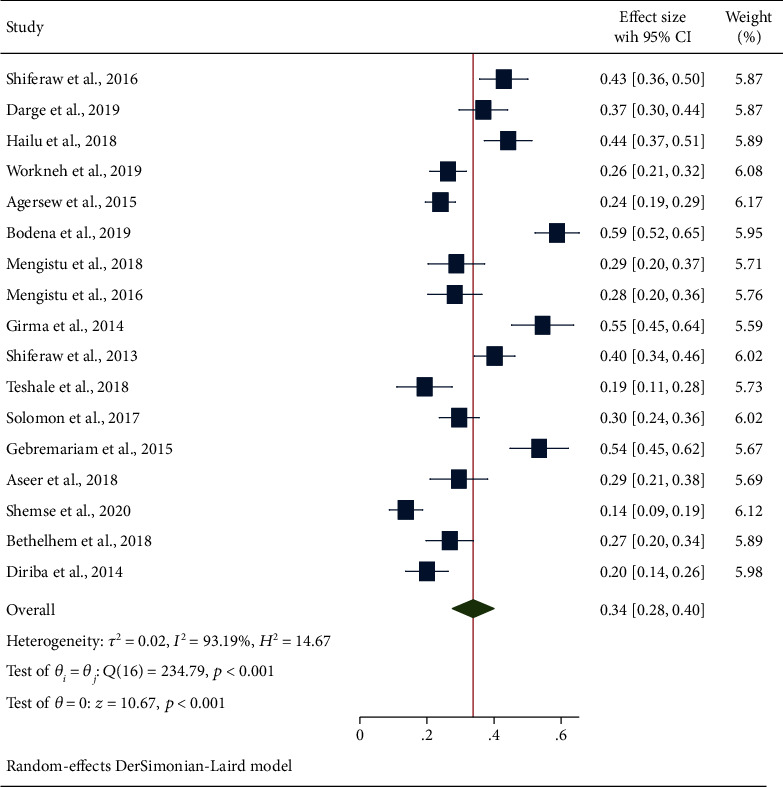
The forest plot showing the prevalence of CoNS from swabs of inanimate surfaces and equipment in Ethiopia.

**Figure 7:**
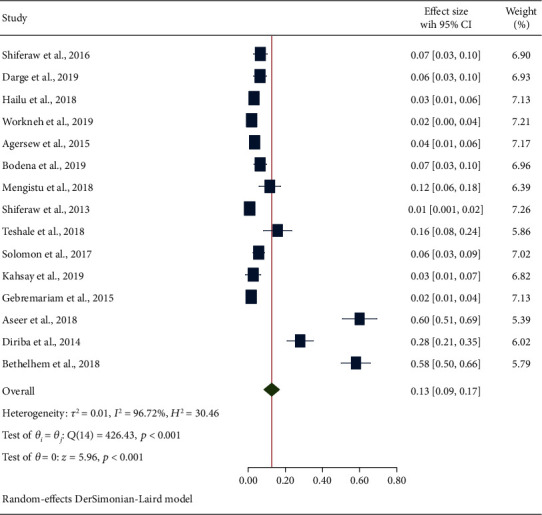
The forest plot showing the prevalence of E. coli from swabs of inanimate surfaces and equipment in Ethiopia.

**Figure 8:**
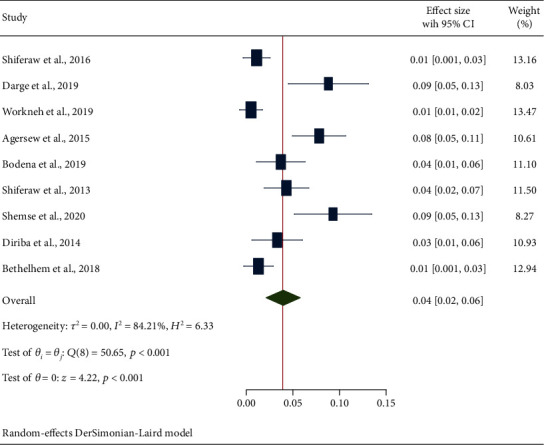
The forest plot showing the prevalence of Citrobacter spp. from swabs of inanimate surfaces and equipment in Ethiopia.

**Figure 9:**
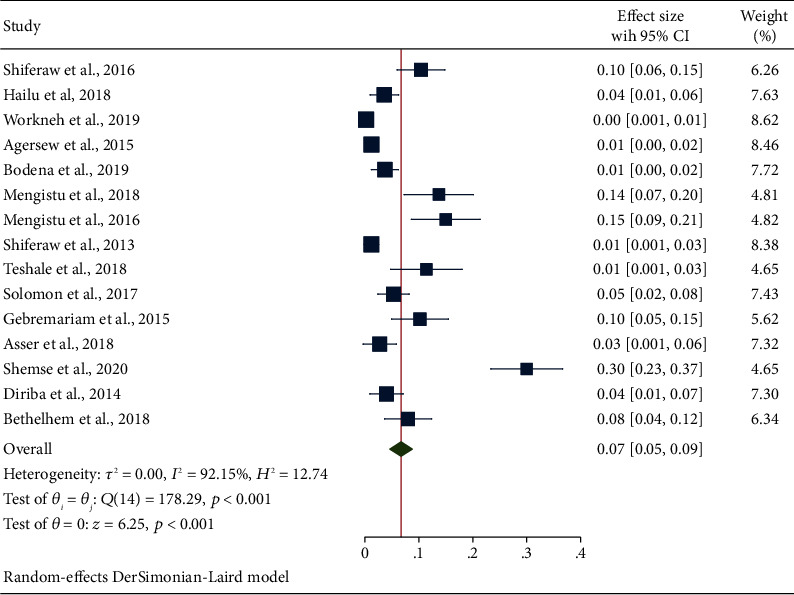
The forest plot depicting the prevalence of P. aeruginosa from swabs of inanimate surfaces and equipment in Ethiopia.

**Figure 10:**
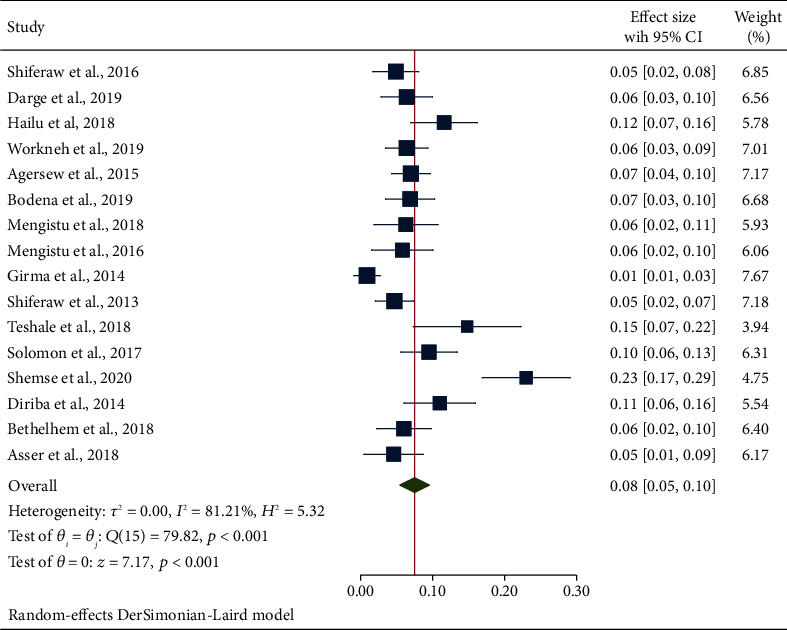
The forest plot depicting the prevalence of K. pneumoniae from swabs of inanimate surfaces and equipment in Ethiopia.

In this meta-analysis, clinically relevant Gram-positive bacterial species (*S. aureus* and CoNS) and Gram-negative bacterial species (*E. coli*, *P. aeruginosa*, *K. pneumoniae*, and *Citrobacter* spp.) were the predominant bacterial species responsible for contaminating inert surfaces and equipment. From the Gram-positive cocci, the pooled prevalence of *S. aureus* obtained from swabs of inanimate surfaces and equipment was 26% (95% CI: 22, 30) which was comparatively lower than the overall prevalence of CONS that was 34% (95% CI: 28, 40). The prevalence of the culture positivity of *S. aureus* (Figure 5) and CoNS is depicted in Figure 6. Concerning Gram-negative aerobic bacilli, the pooled estimates of *E. coli* 13% (95% CI: 9, 17) were comparatively higher than that of *P. aeruginosa* 7% (95% CI: 5, 9) and *K. pneumoniae* 8% (95% CI: 5, 10). The pooled estimate of *E. coli* (Figure 7), *Citrobacter* spp. (Figure 8), *P. aeruginosa* (Figure 9), and *K. pneumoniae* (Figure 10) is already summarized in forest plots depicting the culture positivity of the respective isolates recovered from the swabs.

#### 3.4.4. Third Outcome Measures: Antimicrobial Resistance Patterns

The antimicrobial resistance patterns of the six isolates have shown that isolates were tested against the various antimicrobial agents including penicillin (ampicillin, amoxicillin-clavulanic acid), cephalosporins (ceftriaxone), fluoroquinolones (ciprofloxacin), macrolides (erythromycin), sulfonamides (cotrimoxazole, trimethoprim/sulfamethoxazole), and aminoglycosides (gentamicin). The antimicrobial resistance patterns of the six isolates (*S. aureus*, CoNS, *E. coli*, *P. aeruginosa*, *K. pneumoniae*, and *Citrobacter* spp.) against selected antimicrobial drugs have also been summarized under Table 4. In this regard, the frequency and the cross-ponding percentage calculation were based on the selected nine antimicrobial agents provided that other antimicrobial agents were not in the interest of this study.

Conducting AMR for clinically important pathogens has paramount importance for clinicians to properly treat infections. This can be achieved through the selection of appropriate antimicrobial agents since the drug pattern of the spectrum varies by isolate types. In this study, the pooled estimate of *S. aureus* resistance to ampicillin (AMP) was found at 52% (95% CI: 49, 91). Likewise, *S. aureus* has shown the lowest resistance 11 (95% CI: 4, 18) to gentamicin (GEN) among the tested antimicrobial agents (Table 5).

In the meantime, the prevalence of amoxicillin-clavulanic acid (AMC) resistance CoNS was 71 (95% CI: 53, 87) while the lowest resistance was for cotrimoxazole (SXT), 11 (95% CI: 8, 23). Besides, the pooled prevalence of the Gram-negative isolates was also performed separately based on the selected antimicrobial agents (Table 6). Among the tested drugs, *E. coli* has shown the highest resistance to cotrimoxazole 81% (95% CI: 61, 85) followed by ampicillin 57% (95% CI: 45, 83).

### 3.5. Sensitivity Analysis and Publication Bias

**Figure 11:**
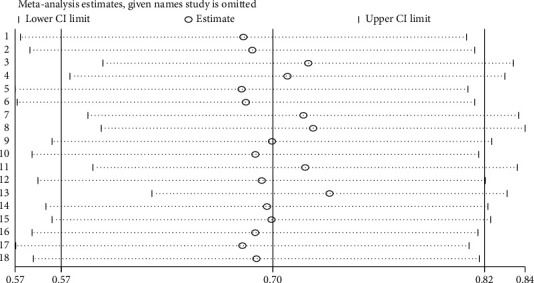
Result of sensitivity analysis of the 18 studies.

Sensitivity analysis was conducted to ensure the stability of the overall effect estimate. The result of sensitivity analyses by using the random-effects model has shown that no single study unduly influenced the overall estimate. Therefore, removing a single study from the analysis did not significantly influence the pooled estimate (Figure 11).

**Figure 12:**
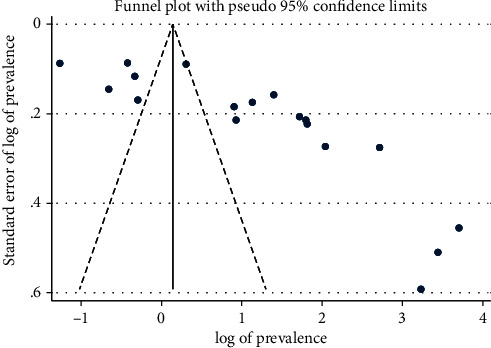
Funnel plot to test the publication bias in the 18 studies.

The presence or absence of publication bias for this study was assessed using a funnel plot and Egger's test. The visual inspection on the funnel plot (Figure 12) suggested the presence of publication bias as revealed by the asymmetrical distribution of the studies. Likewise, the asymmetry of the funnel plot has shown a statistically significant association as evidenced by Egger's test (*p* = 0.006) which in turn declares the presence of small-study effects among the included studies. However, asymmetry in the funnel plots might not be always associated with publication bias [34]. The presence of high heterogeneity among the studies may be one reason for the asymmetry of the funnel plot in this study.

## 4. Discussion

The hospital environment is the potential reservoir for many pathogens responsible for nosocomial infections provided that hospital-acquired infection has remained one of the serious public health problems worldwide [14]. Bacterial contamination of inanimate surfaces and equipment poses a great threat to the cross-transmission of healthcare-associated infections [51]. Adequate infection prevention and control shall be established including effective surface cleaning, disinfecting, and sterilizing of the devices used during and after the patient diagnosis and treatment [2, 27].

According to this systematic review and meta-analysis, the pooled prevalence of bacterial contamination of inanimate surfaces and equipment was found to be 70% (95% CI: 59, 82). The present finding is comparatively lower than the previously conducted different studies across the globe including a study (*n* = 24 swabs) from Egypt [52] that was conducted to investigate microbial contamination of computer keyboards (99.9%) and mice (100%). Likewise, higher results than the current finding such as research findings from Brazil [14] done to assess bacterial contamination of inert hospital surfaces and equipment in critical and noncritical care units with the prevalence of 94.1%; Libya [53, 54] with a contamination rate of 99% and 100%, respectively; Morocco [55] with a contamination rate of 88%; the bacteriological study of electronic devices used by HCW in Rwanda [56] with contamination of 98.53%; a study in Slovakia [57] that was done to assess bacterial contamination of mobile phone and computer keyboard (92%); and the study in the United Kingdom [58] that reported a 95.7% of bacterial contamination of hospital bed-control handsets in a surgical setting were reported. The substantially higher reports of the previous studies as compared to the present findings may be explained due to the difference in the study design [14, 58, 59]; the frequency of cleaning, disinfecting, and sterilizing (use of irradiations) of surfaces and equipment used directly or indirectly for the patient diagnosis and treatment [5, 52]; time of swabbing [60]; awareness of the HCW about microbial contamination of inanimate surfaces and devices [2]; the capability of the pathogen to form biofilm on inanimate surfaces and electronic devices which enables them to survive longer [61]; and variation in the type of microbial reservoir [53]. Moreover, the discrepancy could be due to variation in the study setting, selection of appropriate cleaning and disinfecting products, disinfectant kill time, and existing infection prevention and control strategies [51]. The present finding is comparable with the study conducted on bacterial contamination of an operating theatre in Nigeria [62] with contamination of 78%, a study in Iran [21] on microbial contamination of keyboards and electronic equipment of ICU (*n* = 76) showing a 76% contamination rate of computer keyboards and electronic devices, a study conducted in India [60] to assess the bacterial contamination of stethoscope diaphragms (*n* = 200) sampled from HCWs (79%), a study in Canada [63] on bacterial contamination of surgical loupes (68.75%), and a study in Sri Lanka [64] conducted to assess anesthetist personal mobile phones and wristwatch bacterial contamination during theatre sessions (70%). Also, a lower prevalence of bacterial contamination of inanimate surfaces and equipment compared to the present finding was reported including a study (2009–2015) done in Egypt [65] to assess bacteriology of inanimate surfaces and equipment among selected hospitals which report a prevalence of 25.6% and studies from India [66, 67] aimed at evaluating bacterial contamination from clinical inert environment (*n* = 100) and cell phone of HCW (=30) which report 38% and 42.8% of contamination, respectively. Besides, a study on medical device and equipment surface contamination in three tertiary hospitals (*n* = 1043 swabs) in Baghdad of Iraq [68], another study on microbial contamination of operating theatres and ICU at a specialized hospital in Erbil City of Iraq [69], a study on the bacterial profile from inanimate surfaces and contaminated equipment in ICU at a teaching hospital in Libya [70], another study on the occurrence of bacterial contamination in the operation theatre and related to surgical site infection in Libya [71], a study in Malawi conducted to assess the bacterial profile of toilets and bathroom door handle/knob contamination [15], a study in Palestine to monitor bacterial contamination (*n* = 243 swabs) of the operation theatre [72], and hospital-wide survey of bacterial contamination of point-of-care devices like ultrasound probes in the United States of America [73] had reported a much lower prevalence of 7.86%, 48.3%, 12.5%, 6.7%, 42.6%, 24.7%, and 5.6%, respectively. The discrepancy may be due to the difference in the sample size, study area, study design [14, 59], practices of cleaning and decontaminations [51], facility infection prevention and control [2, 27], type of contaminating isolate [74], and the type of equipment or inanimate surface with respect to the degree of contact [22, 75].

Regarding the contaminating bacterial etiology, two Gram-positive bacterial species (*S. aureus* and CoNS) and four Gram-negative bacterial species (*E. coli*, *P. aeruginosa*, *K. pneumoniae*, and *Citrobacter* spp.) were reported. From the Gram-positive cocci, the CoNS has shown a higher pooled estimate of 34% (95% CI: 28, 40) compared with *S. aureus* 26% (95% CI: 22, 30). In concordant with the present finding, a six-year study conducted in Egypt [65] has revealed that CoNS (32%), followed by *S. aureus* (26%), *K. pneumoniae* (10.6%), and *E.coli* (3%), was the most predominant bacterial species in inanimate surfaces and equipment among referral hospitals. Unlike the current finding, a multicentre study in Brazil [76] has revealed that *S. aureus* was the most common isolate responsible for contaminating inert surfaces (62%) and medical equipment (41%) in the hospital. In the study, in line with our present finding, *P. aeruginosa* (7%) was responsible for contaminating inanimate surfaces and devices. Another study in India [66] has also reported a much lower prevalence of *S. aureus* (6%) and CoNS (5%) compared to our finding. Another study from India [77] has reported a higher prevalence of *P. aeruginosa* (17%) compared to our study but smaller prevalence of *S. aureus* (7%) and *K. pneumoniae* (2%) than the present results. Inconsistent with the present finding, a study in Iran [21] has shown that CoNS (72%) was the most predominant pathogens with a low prevalence of *S. aureus* (6.6%). Opposite to the present finding, a higher prevalence of *E.coli*, *K. pneumoniae*, and *P. aeruginosa* each 30% was reported in Iran [20]. The difference in the distribution of the isolates may be due to the variation in study design [78], contaminant reservoir [76], and study preference on the pathogen's antibiotic resistance from the inert hospital environment or equipment [16] particularly extended-spectrum beta-lactamase-producing bacteria contamination of medical equipment and surfaces [74]. Also, the variation may be explained by the difference in the type of highly and frequently touched surfaces and objects [77], the ability of biofilm formation [61, 77], the absence of best practices to overcome contamination and cross-colonization from potential surface reservoirs [79] which in turn facilitate surfaces of the hospital environment and equipment as possible deposits of resistant bacteria [80], and type of contaminant reservoir [75, 81].

The antimicrobial resistance patterns of medically important isolates have also been identified in this study. In the present study, the prevalence of ampicillin (AMP) resistance *S. aureus* was 52% (95% CI: 49, 91) which is similar to the nationwide study in Brazil [14]. The study also reported that the prevalence of *E. coli* resistance to ceftriaxone (CRO) was 20% which was lower than the present prevalence of *E. coli* resistance to ceftriaxone 37% (95% CI: 16, 57). Other studies from Nigeria 100% [82] and Libya 98% [83] have reported a much higher prevalence of ampicillin- (AMP-) resistant *S. aureus*. Besides, a study in Zimbabwe [84] has revealed that the prevalence of ceftriaxone-resistant *K. pneumoniae* and *E. coli* was 9/13 (69.23%) and 6/11 (54.55%), respectively. The discrepancy might be due to the difference in the study area, the pathogen antimicrobial selective pressure, or aquations of resistance genes [85, 86].

### 4.1. Strength and Limitation of This Study

This meta-analysis has tried to exhaustively search potentially relevant studies across the different regions of Ethiopia. The findings will provide significant clinical implications to healthcare providers to guide empirical therapy. Based on the investigators' knowledge, this systematic review and meta-analysis study is the first in Ethiopia. Consequently, it will offer sufficient information for further studies in the country especially to the HCW regarding the safety of equipment and inert surfaces. On the other side, the major limitation of this study was the lack of studies in some locations of Ethiopia so that the result may not represent a national figure. Moreover, AMR patterns in terms of multidrug-resistant, extended-spectrum beta-lactamase (ESBL), as well as carbapenemase-producing organisms, are not identified in this meta-analysis study.

## 5. Conclusion and Recommendations

In this meta-analysis, the pooled estimate of bacterial contamination of inanimate surfaces and equipment is high (70%). Microbial contamination of the healthcare environment, especially with pathogenic bacterial isolates including Gram-positive cocci (*S. aureus*, CoNS) and Gram-negative bacilli (*E. coli*, *P. aeruginosa*, *K. pneumoniae*, and *Citrobacter* spp.), are responsible for causing HAI. The high prevalence in this study may be associated with a lack of routine practice including disinfecting, cleaning, decontaminating, and sterilizing of inanimate environmental surfaces like medical wards (MW), pediatric wards (PW), orthopaedic wards (OPW), surgical wards (SW), gynecologic wards (GW), emergency wards (EW), intensive care unit (ICU), and neonatal intensive care unit (NICU) as well as medical equipment like a stethoscope, thermometer, sphygmomanometer, ultrasound machine, X-ray machines, magnetic resonance imaging (MRI), and many other HCW fomites. Furthermore, the alarming increase in AMR particularly among the Gram-negative bacterial species demands the use of antimicrobial surfaces to reduce microbial contamination of highly touched healthcare surfaces (beds, door handle, window handle, charts, mobile phones, and cesarian section table). Also, educating HCWs to actively engaged in reducing microbial contamination among highly touched areas or devices is imperative. For example, improving the hand hygiene of HCW will have a great role in reducing cross-contamination of HAIs to and from the patients. Moreover, further studies primarily focusing on the multidrug-resistant bacteria such as extended-spectrum beta-lactamase- (ESBL-) producing Gram-negative bacilli and *Enterobacteriaceae* which are capable of contaminating inanimate environmental surfaces and medical equipment should be investigated to generate local epidemiological data so that the existing infection prevention and control strategies for HAI will be advanced.

## Figures and Tables

**Table 1:** Quality assessment of included studies using JBI's critical appraisal tools.

Studies	9-point Joanna Briggs Institute (JBI) critical appraisal tools										
	Q1	Q2	Q3	Q4	Q5	Q6	Q7	Q8	Q9	Overall score	Include
Shiferaw et al., 2016 [3]	Y	Y	Y	Y	Y	Y	Y	Y	Y	9	✓
Darge et al., 2019 [28]	Y	Y	Y	Y	N	Y	N	Y	Y	7	✓
Hailu et al., 2018 [29]	Y	Y	Y	Y	Y	Y	Y	Y	Y	9	✓
Workneh et al., 2019 [4]	U	Y	Y	Y	Y	Y	Y	Y	Y	8	✓
Agersew et al., 2015 [39]	Y	Y	Y	Y	Y	N	N	Y	Y	7	✓
Bodena et al., 2019 [40]	N	Y	Y	Y	U	Y	Y	N	Y	6	✓
Mengistu et al., 2018 [41]	N	N	Y	Y	N	Y	Y	Y	N	5	✓
Mengistu et al., 2016 [13]	Y	Y	Y	Y	Y	Y	Y	Y	Y	9	✓
Girma et al., 2014 [42]	Y	Y	Y	Y	Y	Y	Y	Y	Y	9	✓
Shiferaw et al., 2013 [24]	N	Y	Y	Y	Y	Y	Y	N	Y	7	✓
Teshale et al., 2018 [43]	Y	Y	Y	N	Y	Y	Y	Y	Y	8	✓
Solomon et al., 2017 [44]	Y	Y	Y	Y	Y	Y	Y	Y	Y	9	✓
Kahsay et al., 2019 [45]	Y	Y	Y	Y	Y	Y	Y	Y	Y	9	✓
Gebremariam et al., 2015 [46]	Y	Y	Y	Y	Y	Y	Y	Y	Y	9	✓
Asser et al., 2018 [47]	Y	N	Y	Y	Y	Y	Y	Y	Y	8	✓
Shemse et al., 2020 [19]	Y	Y	Y	U	Y	Y	N	Y	Y	7	✓
Diriba et al., 2014 [48]	Y	Y	Y	Y	Y	Y	Y	Y	Y	9	✓
Bethelhem et al., 2018 [49]	N	Y	Y	U	Y	Y	N	Y	Y	6	✓

Y: yes; N: no; U: unclear; Q: question. The overall score is calculated by counting the number of Y's in each row (scores of five and above were included in the systematic review and meta-analysis). Q1 = was the sample frame appropriate to address the target population? Q2 = were study participants sampled in an appropriate way? Q3 = was the sample size adequate? Q4 = were the study subjects and the setting described in detail? Q5 = was the data analysis conducted with sufficient coverage of the identified sample? Q6 = were valid methods used for the identification of the condition? Q7 = was the condition measured in a standard, reliable way for all participants? Q8 = was there appropriate statistical analysis? Q9 = was the response rate adequate, and if not, was the low response rate managed appropriately?

**Table 2:** Characteristic of the included articles describing the prevalence of culture-positive bacterial contamination of inanimate surfaces and equipment from the different regions of Ethiopia (2013-2020).

Study	Study period	Study site (region)	Study setting	Wards/units	Equipment	Surfaces/air	Study design	Sampling technique	Number of samples	Sample collection method	Total isolate	Culture positive No. (%)
Shiferaw et al., 2016 [3]	May to August 2013	Adama (Oromia)	Adama Hospital Medical College	SW, GW, NICU, and OR	—	Surface and air of the wards	Cross-sectional	Purposive	78	Settle plate	182	75 (96.2)
Darge et al., 2019 [28]	October 2016 to June 2017	Mekelle (Tigray)	Ayder Comprehensive Specialized Hospital	ICU (adult, pediatric, and neonatal)	Stethoscope, thermometers, sphygmomanometer, mattresses, bedsides, computer, and tables	The surface of the ward	Cross-sectional	Purposive	130	Surface swab	171	115 (88.5)
Hailu et al., 2018 [29]	15 February to 30 April 2017	Bahir Dar (Amhara)	Felege Hiwot Referral Hospital	SW, NICU, ICU, OPW, OR, DW, and GW	—	Surfaces of the wards	Cross-sectional	Purposive	356	Both	190	141 (39.6)
Workneh et al., 2019 [4]	February to April 2017	Bahir Dar (Amhara)	Felege Hiwot Referral Hospital	—	HCW fomites (mobile phones, stethoscope, and white coat)	Surface of the fomites	Cross-sectional	Simple random	422	Surface swab	253	243 (57.6)
Agersew et al., 2015 [39]	April 30 to June 30, 2013	Gondar (Amhara)	University of Gondar Teaching Hospital	—	Computer keyboards and mice	Surfaces of the fomites	Cross-sectional	Simple random	206	Surface swab	344	201 (97.6)
Bodena et al., 2019 [40]	February to March 2018	Harar (Harari)	Hiwot Fana Specialized University Hospital	—	HCW fomites (mobile phones)	Surfaces of the fomite	Cross-sectional	Simple random	226	Surface swab	216	212 (93.8)
Mengistu et al., 2018 [41]	July to September 2017	Hawassa (Sidama)	Hawassa University Comprehensive Specialized Hospital	SW, OR, NICU, OPW, MW, GW, and PW	—	Air of the wards	Cross-sectional	Simple random	96	Settle plate	111	41 (42.7)
Mengistu et al., 2016 [13]	November 2014 to February 2015	Hawassa (Sidama)	Hawassa University Comprehensive Specialized Hospital	ICU, OR	—	Air of the wards	Cross-sectional	Purposive	120	Settle plate	120	41 (34.2)
Girma et al., 2014 [42]	June 15 to October 21, 2011	Jimma (Oromia)	Jimma University Specialized Hospital	—	HCW fomites (mobile phones)	Surface of the fomite	Cross-sectional	Simple random	132	Surface swab	112	94 (71.2)
Shiferaw et al., 2013 [24]	May to September 2011	Jimma (Oromia)	Jimma University Specialized Hospital	—	Stethoscope	Surface of the fomite	Cross-sectional	Simple random	176	Surface swab	256	151 (85.8)
Teshale et al., 2018 [43]	December 1, 2016, to February 30, 2017	Mizan-Tepi (southern Ethiopia)	Mizan-Tepi University Teaching Hospital	EW, MW, GW, PW, SW, and OR	Stethoscope, thermometer, window handle, door handle, bed surfaces, and wall surfaces	The surface of the fomites	Cross-sectional	Purposive	201	Surface swab	88	84 (41.8)
Solomon et al., 2017 [44]	November 2015 to March 2015	Wolaita Sodo (southern Ethiopia)	Wolaita Sodo University Teaching and Referral Hospital	GW, ICU, and OR	—	Surfaces of the wards	Cross-sectional	Purposive	243	Settle plate	226	195 (80.2)
Kahsay et al., 2019 [45]	January to February 2017	Mekelle (Tigray)	Ayder Comprehensive Specialized Hospital	—	Public transport buses	Surface of the buses	Cross-sectional	Purposive	300	Surface swab	66	66 (22)
Gebremariam et al., 2015 [46]	Not specified	Mekelle (Tigray)	Ayder Comprehensive Specialized Hospital	OR	—	Surfaces of the ward	Cross-sectional	Purposive	168	Both	127	127 (75.6)
Asser et al., 2018 [47]	May to June 2018	Arba Minch (southern Ethiopia)	Arba Minch Hospital	SW, NICU, and PW	Stethoscope, thermometer, mobile phones, door handle, bed surfaces, wall surfaces, and white coats	Surfaces of the fomites	Cross-sectional	Purposive	99	Surface swab	109	71 (71.7)
Shemse et al., 2020 [19]	June to September 2018	Addis Ababa (central Ethiopia)	Tikur Anbessa Specialized Teaching Hospital	OR, ICU	Ventilator, lobby, bed, suction machine, and sink	Surface and air of the wards	Cross-sectional	Purposive	164	Surface swab	183	141 (86)
Diriba et al., 2014 [48]	May to August 2011	Hawassa (Sidama)	Hawassa University Comprehensive Specialized Hospital	EW, SW, MW, GW, and PW	—	The surface and air of the wards	Cross-sectional	Purposive	128	Settle plate	153	124 (96.9)
Bethelhem et al., 2018 [49]	Feb 2018 to April 2018	Addis Ababa (central Ethiopia)	12 hospitals in Addis Ababa	—	Radiology parts, X-ray, ultrasound, computed tomography, and magnetic resonance imaging	Surface of the fomites	Cross-sectional	Purposive	178	Surface swab	151	151 (84.8)

ICU: intensive care unit; OR: operating room; HCW: healthcare workers; SW: surgical ward; NICU: neonatal intensive care unit; OPW: orthopaedic ward; DW: dialysis ward; GW: GYN ward; EW: emergency ward; PD: pediatric wards; MW: medical wards.

**Table 3:** Characteristics of included studies reporting the profiles of clinically relevant bacterial species causing inanimate surface and equipment contamination in Ethiopia (2013-2020).

Studies	No. of isolates	Gram-positive bacterial species		Gram-negative bacterial species				
		*S. aureus* No. (%)	CoNS No. (%)	*E. coli* No. (%)	*P. aeruginosa* No. (%)	*K. pneumoniae* No. (%)	*Citrobacter* spp. No. (%)	Others^#^ No. (%)
Shiferaw et al., 2016 [3]	182	37 (20.3)	78 (42.9)	12 (6.6)	19 (10.4)	9 (4.9)	2 (1.1)	25 (14)
Darge et al., 2019 [28]	171	43 (25.1)	63 (36.8)	11 (6.4)	—	11 (6.4)	15 (8.8)	28 (16.4)
Hailu et al., 2018 [29]	190	71 (37.4)	84 (44.2)	6 (3.2)	7 (3.6)	22 (11.6)	—	—
Workneh et al., 2019 [4]	253	81 (19.2)	111 (26.3)	8 (1.9)	1 (0.23)	27 (6.4)	2 (0.5)	23 (9.1)
Agersew et al., 2015 [39]	344	49 (14.2)	83 (24)	12 (3.5)	4 (1.2)	24 (7)	27 (7.8)	145 (42.2)
Bodena et al., 2019 [40]	216	31 (14.4)	127 (58.8)	14 (6.5)	8 (3.7)	15 (6.9)	8 (3.7)	13 (6)
Mengistu et al., 2018 [41]	111	40 (36)	32 (28.8)	—	15 (13.5)	7 (6.3)	—	17 (15.3)
Mengistu et al., 2016 [13]	120	36 (30)	34 (28.3)	14 (11.7)	18 (15)	7 (5.8)	—	11 (9.2)
Girma et al., 2014 [42]	112	33 (29.5)	61 (54.5)	—	—	1 (0.9)	—	17 (15.2)
Shiferaw et al., 2013 [24]	256	79 (30.9)	103 (40.2)	2 (0.8)	3 (1.7)	12 (4.7)	11 (4.3)	46 (18)
Teshale et al., 2018 [43]	88	19 (21.6)	17 (19.3)	14 (15.9)	10 (11.4)	13 (14.8)	—	15 (17)
Solomon et al., 2017 [44]	226	64 (26.3)	72 (29.6)	14 (5.7)	13 (5.3)	23 (9.5)	—	40 (18)
Kahsay et al., 2019 [45]	66	54 (18)	—	8 (2.7)	—	—	—	4 (6.1)
Gebremariam et al., 2015 [46]	127	44 (34.6)	68 (53.5)	2 (1.6)	13 (10.2)	—	—	—
Asser et al., 2018 [47]	109	52 (47.7)	32 (29.5)	8 (7.3)	3 (2.75)	5 (4.6)	—	9 (8.3)
Shemse et al., 2020 [19]	183	38 (20.7)	25 (13.7)	—	55 (30)	42 (23)	17 (9.3)	6 (3.3)
Diriba et al., 2014 [48]	153	15 (9.8)	41 (26.8)	29 (20)	6 (4)	17 (11)	5 (3.3)	40 (26.1)
Bethelhem et al., 2018 [49]	151	47 (31)	30 (20)	8 (5.3)	12 (8)	9 (6)	2 (1.3)	43 (28.5)

—: not reported; CoNS: Coagulase-negative *Staphylococci*. # indicates other pathogens like *Serratia* spp, *Bacillus* spp, *Enterobacter* spp, *Streptococcus agalactiae*, *Enterococcus* spp, *Providencia* spp, *Morganella* spp, and *Salmonella* spp.

**Table 4:** Bacterial pathogens and their antimicrobial resistance patterns isolated from inanimate surfaces and equipment in Ethiopia (2013-2020).

Type of isolate	Study	No. of isolates	Number of isolates resistant to								
			CIP (%)	AMP (%)	AMC (%)	GEN (%)	CRO (%)	SXT (%)	ERY (%)	TE (%)	C (%)
*S. aureus*	Shiferaw et al., 2016	37	2 (5.4)	—	—	12 (32.4)	—	2 (5.4)	19 (51.4)	14 (37.8)	17 (45.9)
	Darge et al., 2019	43	11 (23.9)	10 (21.7)	29 (63)	10 (21.7)	13 (28.2)	—	23 (50)	7 (15.2)	10 (21.7)
	Hailu et al., 2018	71	16 (22.5)	—	16 (22.5)	19 (26.7)	23 (32.4)	35 (49.5)	54 (75.5)	29 (41)	20 (28)
	Workneh et al., 2019	81	1 (1.2)	—	—	14 (17)	—	43 (53.1)	49 (60.5)	40 (49.4)	16 (19.8)
	Agersew et al., 2015	49	4 (9)	41 (74)	39 (80)	10 (21)	1 (3)	29 (60)	11 (23)	34 (70)	35 (77)
	Bodena et al., 2019	31	6 (19.3)	19 (61.3)	8 (25.8)	7 (22.6)	6 (19.3)	20 (64.5)	10 (32.2)	—	10 (32.3)
	Mengistu et al., 2016	36	16 (44.4)	13 (36)	20 (55.6)	—	14 (38.9)	16 (44.4)	33 (47.2)	27 (72.2)	18 (50)
	Mengistu et al., 2018	40	10 (25)	11 (27.5)	—	21 (52.5)	12 (30)	7 (17.5)	24 (60)	26 (65)	13 (32.5)
	Girma et al., 2014	33	11 (33.5)	21 (63.6)	—	7 (21.2)	19 (55.6)	—	33 (100)	33 (100)	—
	Shiferaw et al., 2013	79	14 (17.7)	—	—	21 (26.6)	—	29 (36.2)	22 (27.8)	36 (45.6)	35 (44.3)
	Teshale et al., 2018	19	—	—	—	13 (68.4)	16 (89.5)	—	—	—	—
	Solomon et al., 2017	64	23 (31.9)	—	—	21 (32.8)	—	35 (54.7)	—	—	56 (87.5)
	Kahsay et al., 2019	54	6 (11.1)	—	—	—	17 (31.5)	13 (24.1)	6 (11.1)	3 (5.6)	37 (68.5)
	Gebremariam et al., 2015	44	—	19 (45.2)	—	3 (7.1)	—	—	—	16 (38.1)	10 (23.8)
	Asser et al., 2018	52	12 (23.1)	7 (13.5)	—	3 (5.7)	5 (10)	17 (32.7)	9 (17.3)	—	—
	Shemse et al., 2020	38	5 (13.2)	—	21 (55.3)	—	2 (5.3)	—	3 (8)		1 (2.6)
	Diriba et al., 2014	15	5 (33.3)	—	2 (13.3)	—	1 (6.7)	—	—		1 (2.6)
	Bethelhem et al., 2018	47	—	18 (38.3)	4 (8.5)	12 (25.5)	—	—	4 (8.5)	1 (2.1)	—
CoNS	Shiferaw et al., 2016	78	3 (3.8)	—	—	12 (15.4)	—	9 (11.5)	31 (39.7)	20 (25.6)	35 (44.9)
	Darge et al., 2019	63	9 (13.5)	6 (10.1)	38 (62.7)	6 (10.1)	12 (20.3)	—	30 (50)	8 (11.8)	15 (23.7)
	Hailu et al., 2018	84	20 (24)	—	16 (19)	26 (30.5)	54 (64)	48 (57.7)	59 (70.2)	45 (53.4)	26 (31)
	Workneh et al., 2019	111	7 (6.3)	—	—	17 (15)	—	58 (15.3)	56 (50.5)	61 (55)	28 (25.2)
	Agersew et al., 2015	83	—	61 (73)	63 (76)	39 (80)	5 (7)	53 (64)	7 (8)	62 (74)	47 (57)
	Bodena et al., 2019	127	22 (17.3)	67 (52.7)	34 (26.8)	24 (18.9)	15 (11.8)	80 (63)	39 (30.7)	—	31 (24.4)
	Mengistu et al., 2016	34	12 (35.4)	14 (41.2)	12 (35.3)	—	15 (44.1)	14 (41.2)	19 (55.8)	22 (64.7)	13 (38.3)
	Mengistu et al., 2018	32	16 (50)	13 (40.6)	—	21 (65.6)	13 (40.6)	9 (28.1)	19 (59.4)	25 (78.1)	15 (46.9)
	Girma et al., 2014	—									
	Shiferaw et al., 2013	103	10 (9.7)	—	—	16 (15.5)	—	30 (29.1)	27 (26.2)	33 (41.8)	59 (57.3)
	Teshale et al., 2018	14	—	—	—	2 (14.3)	10 (71)	—	—	—	—
	Solomon et al., 2017	72	25 (39.1)	—	—	35 (48.6)	—	48 (66.7)	—	—	49 (68.1)
	Gebremariam et al., 2015	68	7 (10.3)	30 (44.1)	—	9 (13.2)	—	8 (11.8)	—	10 (11.7)	11 (16.2)
	Asser et al., 2018	32	—	16 (50)	8 (32)	—	(9.4)	—	—	2 (6.3)	—
	Shemse et al., 2020	25	6 (24)	—	9 (36)	2 (8)	—	1 (4)	1 (4)	—	2 (8)
	Diriba et al., 2014	41	8 (19.5)	1 (2.4)	—	—	13 (31.7)	—	3 (7.3)	—	—
	Bethelhem et al., 2018	30	—	9 (30)	—	—	14 (46.7)	—	—	—	3 (10)
*E. coli*	Shiferaw et al., 2016	12	9 (75)	11 (91.7)	—	—	—	5 (41.7)	—	7 (58.3)	7 (58.3)
	Darge et al., 2019	11	2 (18.2)	10 (90.9)	1 (50)	—	8 (72.7)	—	—	3 (27.3)	3 (27.3)
	Hailu et al., 2018	6	—	6 (100)	3 (50)	4 (66.7)	1 (12.7)	6 (100)	—	4 (66.7)	4 (66.7)
	Workneh et al., 2019	8	—	7 (87.5)	—	3 (37.5)	—	7 (87.5)	—	6 (75)	4 (50)
	Agersew et al., 2015	14	—	10 (84)	4 (33)	4 (33)	—	11 (92)	—	—	8 (67)
	Bodena et al., 2019	14	2 (14.3)	11 (78.6)	5 (35.8)	—	4 (28.6)	—	4 (28.6)	—	8 (57.1)
	Mengistu et al., 2016	14	2 (14.2)	2 (14.2)	2 (14.2)	—	2 (14.2)	2 (14.2)	5 (35.7)	—	3 (21.4)
	Shiferaw et al., 2013	2	—	1 (50)	—	1 (50)	1 (50)	—	—	—	—
	Teshale et al., 2018	14	—	10 (71.4%)	—	4 (28.6)	6 (42.8)	—	—	—	—
	Solomon et al., 2017	14	7 (50)	—	—	2 (15.4)	5 (35.6)	5 (35.7)	—	—	—
	Kahsay et al., 2019	8	3 (37.5)	—	—	—	—	13 (24.1)	—	6 (75)	6 (75)
	Gebremariam et al., 2015	2	—	—	—	—	—	1 (50)	—	1 (50)	—
	Asser et al., 2018	8	1 (12.5)	—	4 (50)	2 (25)	—	—	—	—	—
	Diriba et al., 2014	29	—	13 (44.8)	—	8 (27.6)	1 (3.4)	—	—	2 (6.9)	—
	Bethelhem et al., 2018	8	2 (25)	—	3 (37.5)	—	—	—	1 (12.5)	—	—
*P. aeruginosa*	Shiferaw et al., 2016	19	15 (78.9)	—	—	14 (73.7)	9 (75)	6 (31.6)	—	—	—
	Hailu et al., 2018	7	6 (86%)	—	—	2 (28.6)	—	7 (100)	—	5 (71.6)	4 (57.2)
	Workneh et al., 2019	1	—	1 (100)	—	1 (100)	—	1 (100)	—	1 (100)	1 (100)
	Agersew et al., 2015	4	1 (25)	3 (75)	3 (75)	1 (25)	—	3 (75)	—	3 (75)	3 (75)
	Bodena et al., 2019	8	5 (62.5)	8 (100)	8 (100)	1 (12.5)	3 (37.5)	8 (100)	8 (100)	—	4 (50)
	Mengistu et al., 2016	18	4 (22.2)	4 (22.2)	5 (27.7)	—	3 (16.7)	5 (27.7)	7 (38.9)	5 (27.7)	5 (27.7)
	Mengistu et al., 2018	15	3 (89)	6 (82)	—	6 (90)	5 (80.4)	2 (76.7)	9 (60)	15 (100)	6 (78)
	Shiferaw et al., 2013	3	—	1 (50)	3 (100)	3 (100)	3 (100)	3 (100)	—	3 (100)	3 (100)
	Teshale et al., 2018	10	—	4 (40)	—	3 (30)	3 (30)	—	—	—	—
	Solomon et al., 2017	13	8 (61.5)	—	—	8 (61.5)	8 (61.5)	9 (69.2)	—	—	—
	Gebremariam et al., 2015	13		—	—	—	—	—	—	—	—
	Asser et al., 2018	3	—	1 (33.3)	—	—	—	2 (66.7)	—	—	—
	Shemse et al., 2020	55	21 (38.2)	—	10 (18.2)	—	7 (12.7)	—	1 (1.8)	—	5 (9.1)
	Diriba et al., 2014	6	2 (33.3)	—	—	2 (33.3)	—	—	—	—	—
	Bethelhem et al., 2018	12	—	—	4 (33.3)	—	5 (41.7)	—	2 (16.7)	1 (8.3)	—
*K. pneumoniae*	Shiferaw et al., 2016	9	3 (33.3)	11 (91.7)	—	2 (22.2)	5 (55.6)	2 (22.2)	—	5 (55.6)	6 (66.7)
	Darge et al., 2019	1	1 (9)	2 (100)	9 (81.8)	—	6 (54.5)	—	—	3 (27.2)	5 (45.4)
	Hailu et al., 2018	22	5 (23)	12 (54.6%)	5 (23)	9 (41)	9 (41)	8 (36.4)	—	7 (32)	7 (32)
	Workneh et al., 2019	27	1 (3.7)	3 (11.1)	27 (100)	8 (29.6)	5 (18.5)	18 (67)	—	15 (56)	13 (48.1)
	Agersew et al., 2015	21	7 (33)	20 (95)	21 (100)	3 (14)	13 (62)	17 (81)	—	16 (77)	17 (81)
	Bodena et al., 2019	15	—	6 (40) 5	4 (33.3)	4 (21.4)	—	10 (66.6)	7 (46.7)	—	3 (20)
	Mengistu et al., 2016	7	1 (14.3)	2 (28.6)	1 (14.3)	—	—	2 (28.6)	2 (28.6)	3 (42.8)	2 (28.6)
	Mengistu et al., 2018	7	2 (88.6)	1 (74.3)	—	3 (42.9)	2 (78.6)	1 (64.3)	4 (57.1)	6 (85.7)	4 (57.1)
	Shiferaw et al., 2013	12	0	8 (66.7)	—	5 (41.6)	9 (75)	5 (41.7)	—	5 (41.7)	6 (50)
	Teshale et al., 2018	13	—	5 (38.5)	—	3 (23.1)	3 (20)	—	—	—	—
	Asser et al., 2018	5	—	1 (20)	—	—	—	2 (40)	—	—	—
	Shemse et al., 2020	42	16 (38.1)	—	10 (23.8)	—	5 (12)	—	8 (19.5)	—	—
	Diriba et al., 2014	17	—	—	6 (35.3)	—	—	—	—	3 (17.6)	—
	Bethelhem et al., 2018	9	3 (33.3)	1 (11.1)	—	—	—	—	—	—	3 (33.3)
*Citrobacter* spp.	Darge et al., 2019	15	1 (6.6)	2 (80)	13 (86.6)	—	—	—	—	—	—
	Agersew et al., 2015	27	2 (29)	27 (100)	27 (100)	6 (21)	20 (75)	20 (75)	—	20 (75)	20 (75)
	Asser et al., 2018	9	4 (44.4)	—	2 (22.2)	—	—	1 (11.1)	—	—	—
	Shemse et al., 2020	6	—	3 (50)	—	—	1 (16.7)	—	—	—	—
	Diriba et al., 2014	40	17 (42.5)	—	6 (15)	3 (7.5)	—	11 (27.5)	—	9 (22.5)	1 (2.5)
	Bethelhem et al., 2018	43	—	19 (44.2)	—	15 (34.9)	3 (7)	—	5 (11.6)	—	—

—: not tested; AMP: ampicillin; AMC: amoxicillin-clavulanic acid; SXT: cotrimoxazole; CRO: ceftriaxone; CIP: ciprofloxacin; GEN: gentamicin; ERY: erythromycin; TE: tetracycline; C: chloramphenicol; CoNS: Coagulase-negative *Staphylococci*.

**Table 5:** Pooled estimated antimicrobial resistance among clinically important Gram-positive bacterial species recovered from swab samples of surfaces and equipment in Ethiopia (2013-2020).

Antimicrobial agents	*S. aureus*		CoNS	
	Pooled estimate (95% CI)	*I* ^2^ (%)	Pooled estimate (95% CI)	*I* ^2^ (%)
Ampicillin (AMP)	0.52 (0.49, 0.91)	97.64	0.62 (0.34, 0.90)	93.05
Amoxicillin-clavulanic acid (AMC)	0.23 (0.21, 0.40)	87.45	0.71 (0.53, 0.87)	98.85
Cotrimoxazole (SXT)	0.45 (0.32, 0.65)	94.02	0.11 (0.08, 0.23)	72.38
Ceftriaxone (CRO)	0.36 (0.11, 0.55)	87.34	0.39 (0.20, 0.55)	88.51
Ciprofloxacin (CIP)	0.34 (0.22, 0.46)	86.61	0.50 (0.33, 0.70)	95.21
Gentamicin (GEN)	0.11 (0.04, 0.18)	84.11	0.42 (0.24, 0.48)	90.05
Erythromycin (ERY)	0.49 (0.31, 0.68)	97.15	0.28 (0.17, 0.50)	89.16
Tetracycline (TE)	0.18 (0.13, 0.34)	84.89	0.13 (0.09, 0.37)	82.09
Chloramphenicol (C)	0.21 (0.12, 0.46)	80.32	0.32 (0.23, 0.52)	87.54

CoNS: Coagulase-negative *Staphylococci*.

**Table 6:** Pooled estimated antimicrobial resistance among clinically important Gram-negative bacterial species recovered from swab samples of surfaces and equipment in Ethiopia (2013-2020).

Antimicrobial agents	*E. coli*		*P. aeruginosa*		*K. pneumoniae*		*Citrobacter* spp.	
	Pooled estimate (95% CI)	*I* ^2^ (%)	Pooled estimate (95% CI)	*I* ^2^ (%)	Pooled estimate (95% CI)	*I* ^2^ (%)	Pooled estimate (95% CI)	*I* ^2^ (%)
Ampicillin (AMP)	0.57 (0.45, 0.83)	55.17	0.77 (0.58, 0.82)	<0.001	0.80 (0.78, 0.92)	57.67	0.78 (0.57, 0.83)	96.81
Amoxicillin-clavulanic acid (AMC)	0.50 (0.40, 0.73)	82.58	0.68 (0.63, 0.89)	64.87	0.63 (0.51, 0.83)	88.01	0.32 (0.36, 0.54)	45.01
Cotrimoxazole (SXT)	0.81 (0.61, 0.85)	79.03	0.95 (0.82, 0.91)	<0.001	0.88 (0.83, 0.91)	64.27	0.78 (0.60, 0.85)	49.67
Ceftriaxone (CRO)	0.37 (0.16, 0.57)	86.09	0.14 (0.07, 0.24)	87.53	0.24 (0.15, 0.46)	88.50	0.12 (0.06, 0.19)	83.68
Ciprofloxacin (CIP)	0.44 (0.31, 0.50)	89.01	0.55 (0.33, 0.81)	90.86	0.57 (0.39, 0.75)	92.02	0.33 (0.24, 0.66)	92.14
Gentamicin (GEN)	0.53 (0.33, 0.57)	85.91	0.76 (0.58, 0.85)	71.81	0.64 (0.51, 0.80)	86.48	0.56 (0.39, 0.72)	84.29
Erythromycin (ERY)	0.24 (0.16, 0.33)	83.09	0.13 (0.11, 0.24)	76.47	0.31 (0.20, 0.53)	89.66	0.11 (0.12, 0.37)	64.41
Tetracycline (TE)	0.44 (0.20, 0.71)	78.91	0.16 (0.08, 0.35)	88.81	0.22 (0.16, 0.39)	80.68	0.26 (0.13, 0.62)	68.29
Chloramphenicol (C)	0.21 (0.16, 0.32)	86.03	0.14 (0.07, 0.43)	72.11	0.18 (0.09, 0.77)	93.68	0.16 (0.13, 0.92)	91.39

## Data Availability

All the data used in this article are presented within the manuscript and additional file without restriction.

## Abbreviations

CoNS: Coagulase-negative *Staphylococci*
HAI: Hospital-acquired infections
HCW: Healthcare workers
ICU: Intensive care unit
JBI: Joanna Briggs Institute
MDR: Multidrug-resistant.

## Supplementary Materials

Supplementary Materials Table S1: PRISMA 2009 checklist. Table S2: bacterial pathogens and their antimicrobial resistance patterns isolated from inanimate surfaces and equipment in Ethiopia (2013-2019).
